# Improvement of Learning and Memory by Elevating Brain D-Aspartate in a Mouse Model of Fragile X Syndrome

**DOI:** 10.1007/s12035-023-03438-0

**Published:** 2023-07-15

**Authors:** Yu-Jiao Li, Kun Zhang, Ting Sun, Yan-Yan Guo, Qi Yang, Shui-Bing Liu, Yu-Mei Wu, Ming-Gao Zhao

**Affiliations:** 1 Department of Pharmacy, Tangdu Hospital, Fourth Military Medical University, Xi’an, 710038 Shaanxi Province China; 2 Department of Pharmacy, General Hospital of Eastern Theater Command/Jinling Hospital, Medical School of Nanjing University, Nanjing, 210002 China; 3 Department of Pharmacology, School of Pharmacy, Fourth Military Medical University, Xi’an, 710032 Shaanxi Province China

**Keywords:** Fragile X syndrome, D-Aspartate, Long-term potentiation, Fear memory, mGluR5

## Abstract

Fragile X syndrome (FXS) is an inherited human mental retardation that arises from expansion of a CGG repeat in the *Fmr1* gene, causing loss of the fragile X mental retardation protein (FMRP). It is reported that N-methyl-D-aspartate receptor (NMDAR)-mediated facilitation of long-term potentiation (LTP) and fear memory are impaired in *Fmr1* knockout (KO) mice. In this study, biological, pharmacological, and electrophysiological techniques were performed to determine the roles of D-aspartate (D-Asp), a modulator of NMDAR, and its metabolizing enzyme D-aspartate oxidase (DDO) in *Fmr1* KO mice. Levels of D-Asp were decreased in the medial prefrontal cortex (mPFC); however, the levels of its metabolizing enzyme DDO were increased. Electrophysiological recordings indicated that oral drinking of D-Asp recovered LTP induction in mPFC from *Fmr1* KO mice. Moreover, chronic oral administration of D-Asp reversed behavioral deficits of cognition and locomotor coordination in *Fmr1* KO mice. The therapeutic action of D-Asp was partially through regulating functions of NMDARs and mGluR5/mTOR/4E-BP signaling pathways. In conclusion, supplement of D-Asp may benefit for synaptic plasticity and behaviors in *Fmr1* KO mice and offer a potential therapeutic strategy for FXS.

## Introduction

Fragile X syndrome (FXS) is a common, inherited mental retardation, which is been scrutinized as a leading genetic cause of autism spectrum disorders [1]. This neurodevelopmental disorder is caused by a CGG repeat across the 5′-non-coding region of the fragile X mental retardation 1 (*Fmr1*) gene, which leads to the loss of fragile X mental retardation protein (FMRP). FMRP is reported to regulate the expression of proteins via interactions with up to 4% of mRNAs in the mammalian brain [2–4]. Thus, loss of FMRP leads to dysregulation of protein synthesis and localization, causing behavioral and cognitive dysfunctions including social interaction deficits, impaired novel object recognition, and behavioral inflexibility in FXS and animal models [5, 6]. The underlying mechanisms of behavioral and cognitive dysfunctions in FXS include the mGluR theory [7], dendritic spine development disorder [8], and serotonin dysregulation [9]. Clinical trials for FXS treatment include mGlu5 inhibitors, GSK3β inhibitors, and antioxidants, but therapeutic effects of these trials are unimpressive. Thus, subtle mechanisms behind FXS are deserved to elucidate for better therapeutic options.

N-methyl-D-aspartate receptors (NMDARs) are glutamate-gated ion channels which are critical for neuronal communication and synaptic plasticity [10], especially for induction and expression of long-term potentiation (LTP) [11]. Previous work indicates that hypofunction of NMDAR in the dentate gyrus leads to the impairment in NMDARs-dependent synaptic plasticity in adult *Fmr1* knockout (KO) mice [12]. Thus, NMDAR regulators, including glycine and D-serine, could be potential treatments for FXS [13]. D-aspartate (D-Asp) is a free D-amino acid found in the mammalian brain with a temporally dependent concentration based on postnatal expression of its metabolizing enzyme D-aspartate oxidase (DDO). The concentration of D-Asp fluctuates over a lifetime, increasing during embryonic and perinatal periods and decreasing during adulthood [14]. D-Asp is one of the NMDAR co-agonists; augmented D-Asp content is able to suppress long-term depression (LTD) in the striatum, and to increase NMDAR-dependent hippocampal LTP and spatial memory [15].

The D-aspartate oxidase (DDO) gene encodes the enzyme responsible for the catabolism of D-aspartate, an atypical amino acid enriched in the mammalian brain and acting as an endogenous NMDA receptor agonist [16]. D-Asp is important to neurogenesis, learning, and neuropathology. Increased D-Asp in the brain rescues hippocampal age-related synaptic plasticity deterioration in mice [17]. Moreover, brain D-Asp can be increased by exogenous D-Asp, so it may hold promise as a therapy.

Here, we performed biological, pharmacological, and electrophysiological experiments to test the effects of D-Asp supplement on the behaviors of *Fmr1* KO mice. We found lower levels of D-Asp but higher DDO expression in the medial prefrontal cortex (mPFC) of *Fmr1* KO mice compared with wild-type (WT) mice. Chronic treatment of *Fmr1* KO mice with oral D-Asp alleviated LTP deficiency and related signaling pathways in FXS mice. Furthermore, D-Asp rescued behavioral impairments, including learning and memory deficits and motor coordination disability of FXS mice. Thus, D-Asp may benefit for the treatment of FXS.

## Materials and Methods

### Materials

D-Asp (purity ≥ 99%, HPLC), N-Acetyl-L-cysteine, DHPG, and β-actin antibody were purchased from Sigma-Aldrich (St. Louis, MO, USA). The chromatographic column (ODS Hypersil, 250 × 4.6 mm, 5 μm) was got from Thermo Fisher Scientific (Waltham, MA, USA). Neurobasal medium, B27, glutamine, and fetal bovine serum (FBS) were provided by Invitrogen (Carlsbad, CA, USA). Dulbecco’s Modified Eagle’s Medium/High Glucose (DMEM/high glucose) was purchased from Hyclone (Logan, UT, USA). Antibodies for p-mTOR, mTOR, p-AKT, AKT, p-4E-BP, and 4E-BP were purchased from Cell Signaling Technology (Danvers, MA, USA). Antibodies for DDO, GluN1, GluN2A, GluN2B, GluR1, p-GluR1-S831, and p-GluR1-S845 were purchased from Abcam (Cambridge, MA, USA). All secondary antibodies conjugated with horseradish peroxidase (HRP) were purchased from Santa Cruz Biotechnology (Santa Cruz, CA, USA). Alexa Fluor 488 goat anti-rabbit IgG and goat anti-rabbit IgG Cy3 conjugated were purchased from Molecular Probes (Eugene, OR, USA). M-PER protein extraction buffer, BCA Kit, and enhanced chemiluminescent solution (ECL) were obtained from Pierce (Pierce, Rockford, IL, USA). PVDF membrane was purchased from Roche (Mannheim, Germany).

### Animals

*Fmr1* KO (FVB.129P2-Fmr1tm1Cgr/J; stock #4624) and control [FVB.129P2-Pde6b+ Tyrc-ch/AntJ; stock #4828; hereafter referred to as wild-type (WT)] strain mice were obtained from the Jackson Laboratory. D-Asp was delivered in drinking water at the concentration of 20 mM to pregnant Fmr1 WT and KO mice until the offsprings were isolated to new cages on postnatal 20 day (P20). Then, the offsprings were continually delivered D-Asp drinking water until 8 weeks old. Then thirty-six male offspring mice were used for behavioral and electrophysiological experiments. All animals were housed in groups in standard cages (29 cm × 17.5 cm × 12.5 cm) at constant temperature (22 ± 1°C) and maintained on a 12 h/12 h light/dark cycle, with food and water ad libitum. The experimental procedures were approved by the Institutional Animal Care and Use Committee of The Forth Military Medical University.

### Detection of D-Asp

The mPFC was dissected and stored at −80°C for further detection. Concentration of D-Asp was detected by high-performance liquid chromatography (HPLC) analysis, based on the diastereomeric separation of D-Asp from the L-form (L-Asp) and other L-amino acids, as previously described [18]. Briefly, the mobile phase was consisted of 50 mM ammonium acetate/methanol (96:4), and the flow rate was 1mL/min. UV detector was used to quantify and determine D-Asp content.

### Primary Culture of Cortical Neurons and Treatments

Primary cortical neurons were isolated from the brain of E_15_-E_16_ C57BL/6 mouse embryos as previous described [19]. Briefly, the prefrontal cortex was dissected, minced, and incubated with 0.25% trypsin in Ca^2+^ and Mg^2+^-free Hank’s balanced salt solution for 20 min at 37°C. Then, the cortices were washed in DMEM supplemented with 10% FBS to stop trypsin activity and further dissociated by trituration. The single cell suspension was cultured onto 6-well plates grown in Neurobasal medium supplemented with B27, 0.5 mM glutamine, 100 U/ml penicillin, and 100 U/ml streptomycin. Half of the medium was changed every 2 days, and neurons were used in the experiments at 14th day in vitro (DIV 14), the time required for maturation of cortical neurons. Neurons were treated with DHPG, an mGluR5 selective agonist, at 100 μM for 5 min. Some of neurons were pretreated with D-Asp (10 μM) or MPEP (an mGlu5 receptor antagonist, 50 μM) for 60 min.

### Western Blot Analysis

Western blot analysis was performed as described previously [20]. Protein concentration was determined using a BCA Kit (Pierce, Rockford, IL, USA). Equal amounts of total protein (50 μg) for each sample were loaded onto 10% polyacrylamide gels. Proteins were separated by SDS-PAGE and transferred to PVDF membranes. Membranes were blocked for 1 h with 5% non-fat milk in Tris-Phosphate buffer containing 0.05% Tween 20 (TBS·T) and immunoblotted overnight at 4°C using selective antibodies against DDO (1:1000), p-GluR1-831 (1:1000), p-GluR1-S845(1:1000), GluR1 (1:1000), GluN1 (1:1000), GluN2A (1:400), GluN2B (1:1000), p-mTOR (1:1000), mTOR (1:1000), p-AKT (1:1000), AKT (1:1000), p-4E-BP (1:1000), 4E-BP (1:1000), and β-actin (1:10000) served as loading control. After three washes for 10 min with TBS·T, membranes were further incubated with HRP-conjugated secondary antibodies for 1–2 h and followed by four TBS·T washes, and bands were visualized using a Tanon5200 imager (Shanghai, China).

### Immunohistochemistry Staining

Brains were fixed with 4% paraformaldehyde. Coronal frozen sections of 50 μm containing the mPFC were harvested. Brain slices were punched out in PBS with 0.3% Triton X-100 and blocked in 2% bovine serum albumin. Subsequently, slices were incubated with rabbit anti-DDO antibody (1:100) at 4 °C overnight, followed by goat anti-rabbit IgG Alexa Fluor 488 (1:200) for 1 h at 25 °C. All antibodies were diluted in PBS with 0.1% Triton X-100 and 2% bovine serum albumin. Nuclei were stained with Hoechst33258.

### Multi-channel Field Potential Recordings

The general procedures for field potential recordings using 64-channel recording system (MED64, Panasonic Alpha-Med Sciences, Japan) were similar to those described previously [19]. Briefly, mice were anesthetized with gaseous isoflurane and decapitated. Brain was rapidly removed and immersed into a cold bath of oxygenated (equilibrated with 95% O_2_ and 5% CO_2_) ACSF containing (in mM): NaCl 124, KCl 2.5, NaH_2_PO_4_ 1.0, MgSO_4_ 1, CaCl_2_ 2, NaHCO_3_ 25 and glucose 10, pH 7.35–7.45. Coronal mPFC slices (300 μm) were obtained and transferred to an incubation chamber continuously perfused for 1–2 h. After incubation, slices were placed on a MED64 probe (MED-P515A, 8 × 8 array, interpolar distance = 150 μm; Panasonic Alpha-Med Sciences) covering most of the 64 electrodes. Electrical stimulation was delivered by one channel located within the deep layer VI of the mPFC, and evoked fEPSPs were monitored and recorded from the other 63 channels. Baseline responses were evoked at 0.017 Hz for at least 30 min before LTP was induced by the theta burst-stimulation (TBS) protocol with no change in stimulation intensity, consisting of five train bursts with four pulses at 100 Hz, at 200 ms interval; repeated five times at intervals of 10 s. The magnitude of potentiation was determined by measuring the changes in fEPSP slope for about 3 h. Drugs were bath applied to observe their actions on the maintenance phase of LTP 20 min before TBS stimulus. In each experiment, 6–8 channels were selected for analysis to get reliable data.

### Locomotor Activity Test

Locomotor activity test was performed as previously described [21]. It was carried out in a square box (30 cm × 30 cm × 30 cm) with clear Plexiglas walls and floor, and placed inside an isolation chamber with dim illumination and a fan. Mice were placed in the center of the box, and activity was measured for 15 min. Mice were videotaped using a camera fixed above the box and analyzed with a video-tracking system.

### Trace Fear Conditioning

Trace fear conditioning was conducted in an isolated shock chamber (Gemini, San Diego Instruments, San Diego, CA) as previously described [19]. The CS (conditioned stimulus) used was an 80 dB white noise, delivered for 15 s, and the US (unconditioned stimulus) was a 0.7 mA scrambled footshock for 0.5 s. On the training day, mice were acclimated for 60 s and were presented with 10 CS-trace-US intertrial interval (ITI) trials (ITI, 210 s). After training, mice were returned to the homecages. One day later, mice were acclimated for 60 s followed by 10 CS-ITI trails (ITI, 210 s) in a novel chamber to test for trace fear memory. Both training and testing sessions were videotaped and analyzed ethologically by an experimenter blind to treatment.

### Rotarod Test

Motor learning was tested using an accelerating rotarod set-up (Shanghai Jiliang, Shanghai, China). Before the test, mice were habituated to the rotating drum by placing them on the drum at the slowest speed for 3 min. Then, mice were tested on the drum accelerated from 4 to 42 rpm over a period of 5 min. Falling latency on the rod was measured. Mice underwent four trials each day for 2 consecutive days with an inter trial interval (ITI) of 30 min.

### Data Analysis

Results were expressed as mean ± SEM. Unpaired Student’s *t*-test was used to test difference between the two groups. Statistical comparisons over two groups were performed using one- or two-way ANOVA and the Student-Newman-Keuls test for post hoc comparisons. In all cases, *P* < 0.05 was considered statistically significant.

## Results

### Lower Levels of D-Asp in mPFC of *Fmr1* KO Mice

**Fig. 1:**
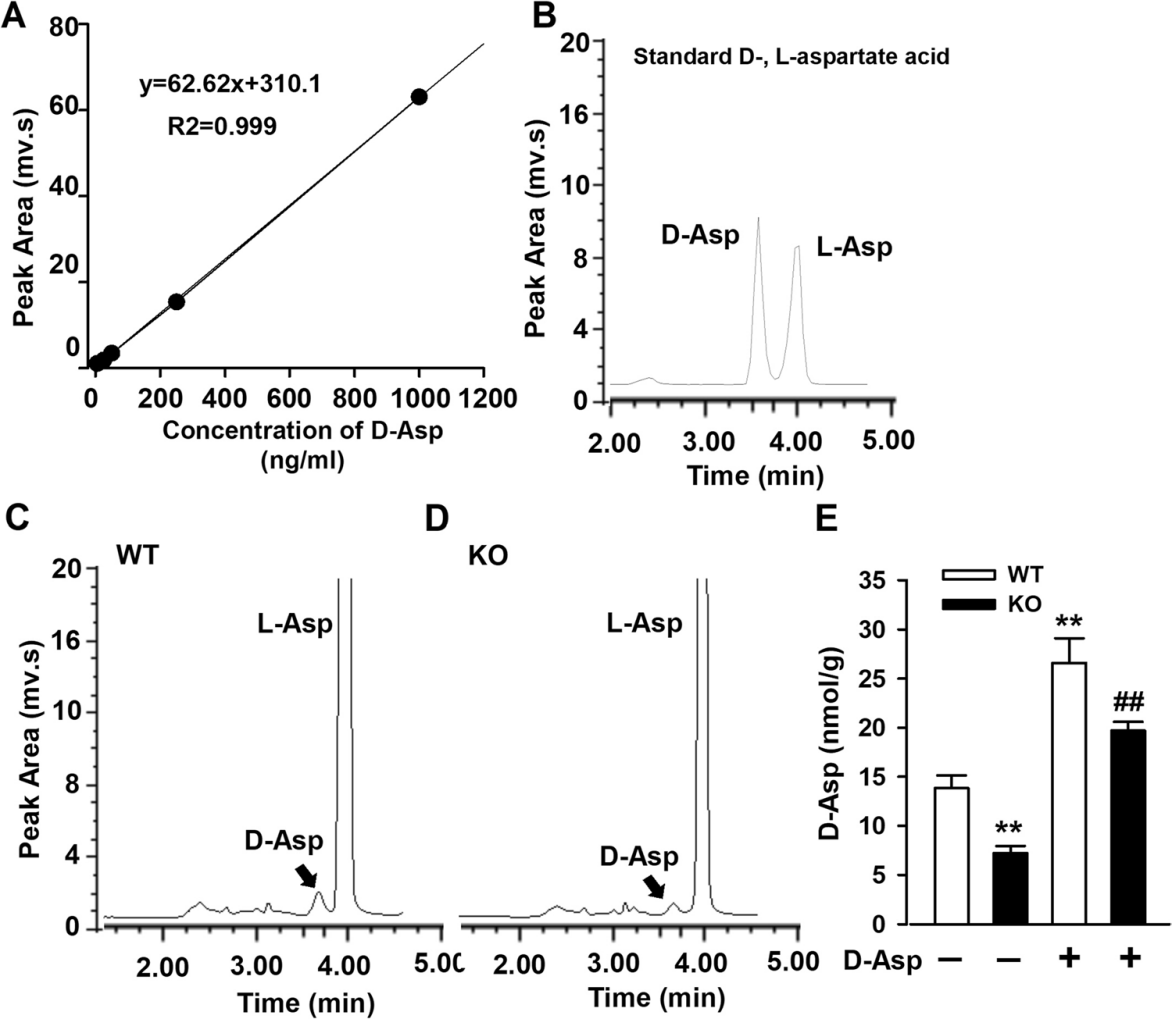
Concentration of D-Asp in mouse brain. **A** Standard curve of D-Asp. **B** Representative HPLC samples of D-Asp (5 μg/ml) and L-Asp (5 μg/ml) with the retention time at 3.8 min and 4.0 min respectively. **C** Typical example of HPLC analysis of D-Asp from *Fmr1* WT mouse brain at 3.8 min. **D** Typical example of HPLC analysis of D-Asp from *Fmr1* KO mouse brain at 3.8 min. **E** Levels of D-Asp in mPFC after D-Asp (20 mM) was added in drinking water. *n* = 6 in each group. **P < 0.01 vs WT control mice; ^##^*P* < 0.01 vs KO control mice with two-way ANOVA-test

Impairments of NMDAR-dependent synaptic plasticity are evident in *Fmr1* KO mice in the dentate gyrus [12] and anterior cingulate cortex (ACC) [22], and D-Asp acting as an endogenous NMDA receptor agonist. To explore the role of D-Asp with regard to FXS pathologic changes, D-Asp was measured in the mPFC area of WT and *Fmr1* KO mice. HPLC was used to separate and quantify D-Asp, L-Asp, and other amino acids as reported previously [18]. Chromatograms clearly showed a visible peak of D-Asp at 3.8 min, which was consistent with D-Asp standards (Fig. 1A and B). HPLC analysis confirmed that D-Asp was significantly lower in mPFC of *Fmr1* KO mice compared to the WT mice at postnatal 8 weeks (7.21 ± 0.74% nmol/g *vs* 13.8 ± 1.33% nmol/g; Fig. 1C and D). D-Asp (20 mM) was delivered in drinking water to pregnant mice until offsprings were 8 weeks old. Oral administration of exogenous D-amino acid replenished brain D-Asp according to a previous report [23]. D-Asp increased ~2-fold in mPFC from offspring at postnatal 8 weeks in both genotypes (WT: 26.6 ± 2.52% nmol/g; KO: 19.7 ± 0.97% nmol/g; Fig. 1E). It indicates that D-Asp in drinking water is an available way to supplement brain D-Asp.

### Higher Levels of DDO in mPFC of Fmr1 KO Mice

**Fig. 2:**
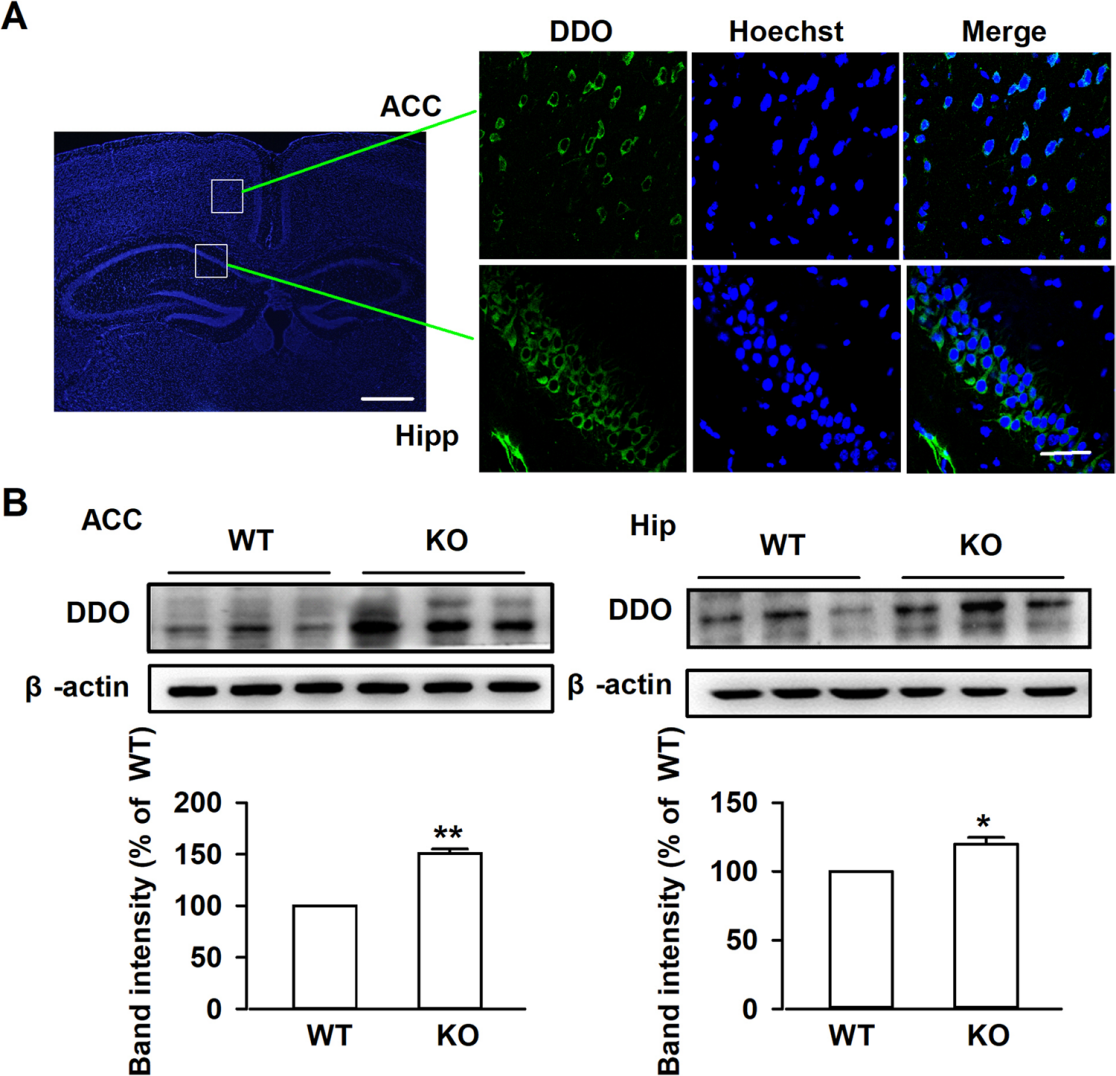
Levels of DDO in mPFC. **A** Representative fluorescence images of DDO and Hoechst double staining. Left panel, scale bar = 500 μm; right panel, scale bar = 50 μm. **B** Western blot analysis for DDO in the mPFC and Hippocampus from *Fmr1* WT and KO mice. *n* = 6 in each group. **P < 0.01 vs WT control mice with unpaired student *t*-test

DDO is the only known enzyme responsible for D-Asp degradation [23]. To investigate the reason for decreased D-Asp in *Fmr1* KO mice; we measured levels of DDO in the brain tissues. DDO was highly expressed in mPFC and hippocampus (Fig. 2A), and the expression was higher in mPFC of KO mice as compared to WT mice. Western blot indicated higher levels of DDO in mPFC and hippocampus of *Fmr1* KO mice (WT: 100 ± 3.24%; KO: 150.8 ± 4.32%; Fig. 2B), suggesting that loss of FMRP may affects the DDO expression in *Fmr1* KO mice.

### Lower Levels of NMDA Receptors in mPFC of Fmr1 KO Mice

**Fig. 3:**
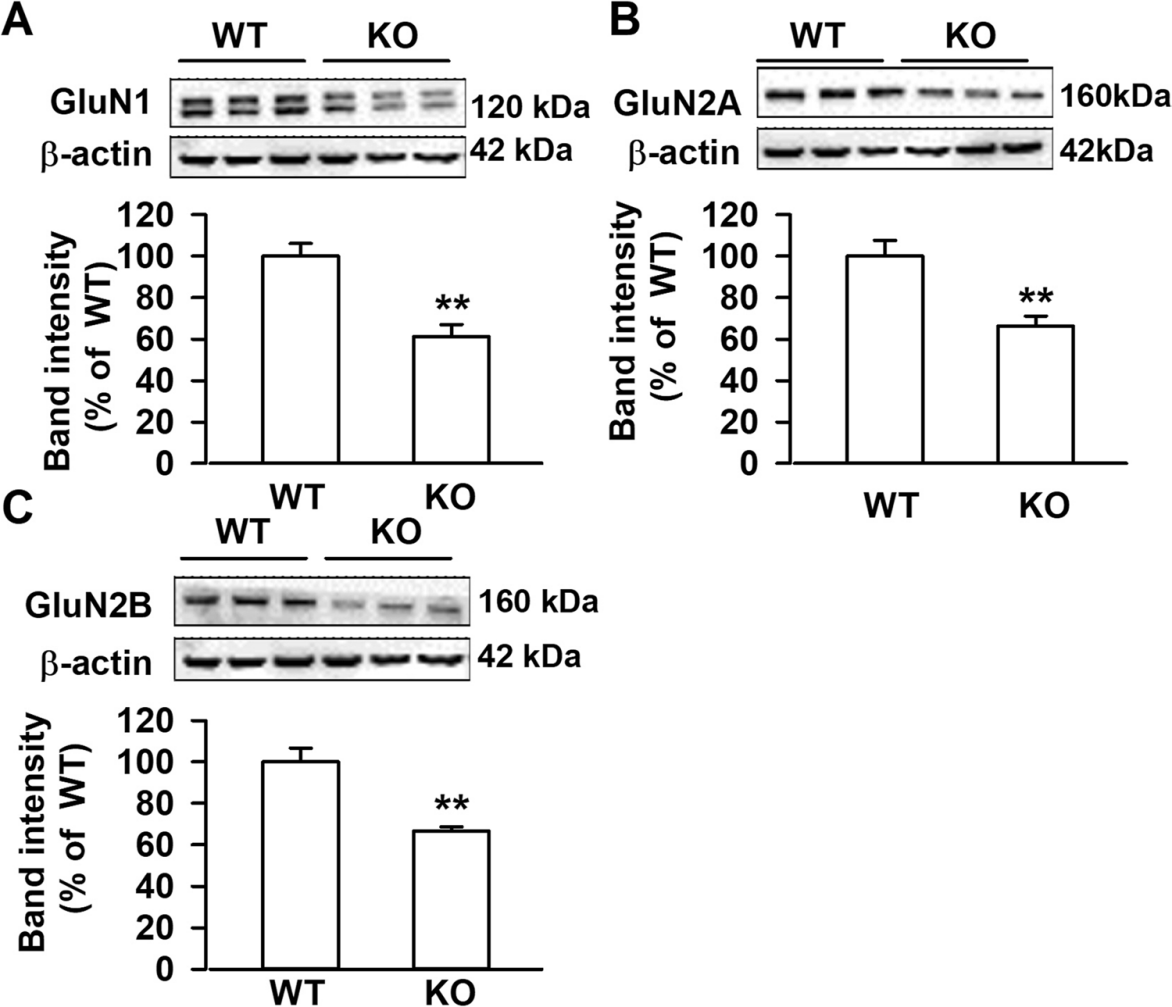
NMDARs expression in mPFC. Western blots were performed with 50 μg of total protein. Significant decrease in levels of GluN1 (**A**), GluN2A (**B**), and GluN2B (**C**) subunit in mPFC of *Fmr1* KO mice as compared to WT mice. *n* = 6 in each group, **P < 0.01 vs WT control mice with unpaired Student’s *t*-test

D-Asp is a NMDAR co-agonist [15]. However, we found impaired NMDAR-mediated LTP in the ACC of *Fmr1* KO mice [22]. Thus, we detected the levels of NMDAR in this brain region. Western blot revealed that protein levels of NMDAR in mPFC, including three critical subunits GluN1, GluN2A, and GluN2B, were lower in KO mice as compared to WT mice (Fig. 3). This is consistent with previous report [24].

### Lower Levels of GluA1 Phosphorylation at Serine-831 in mPFC of Fmr1 KO Mice

**Fig. 4:**
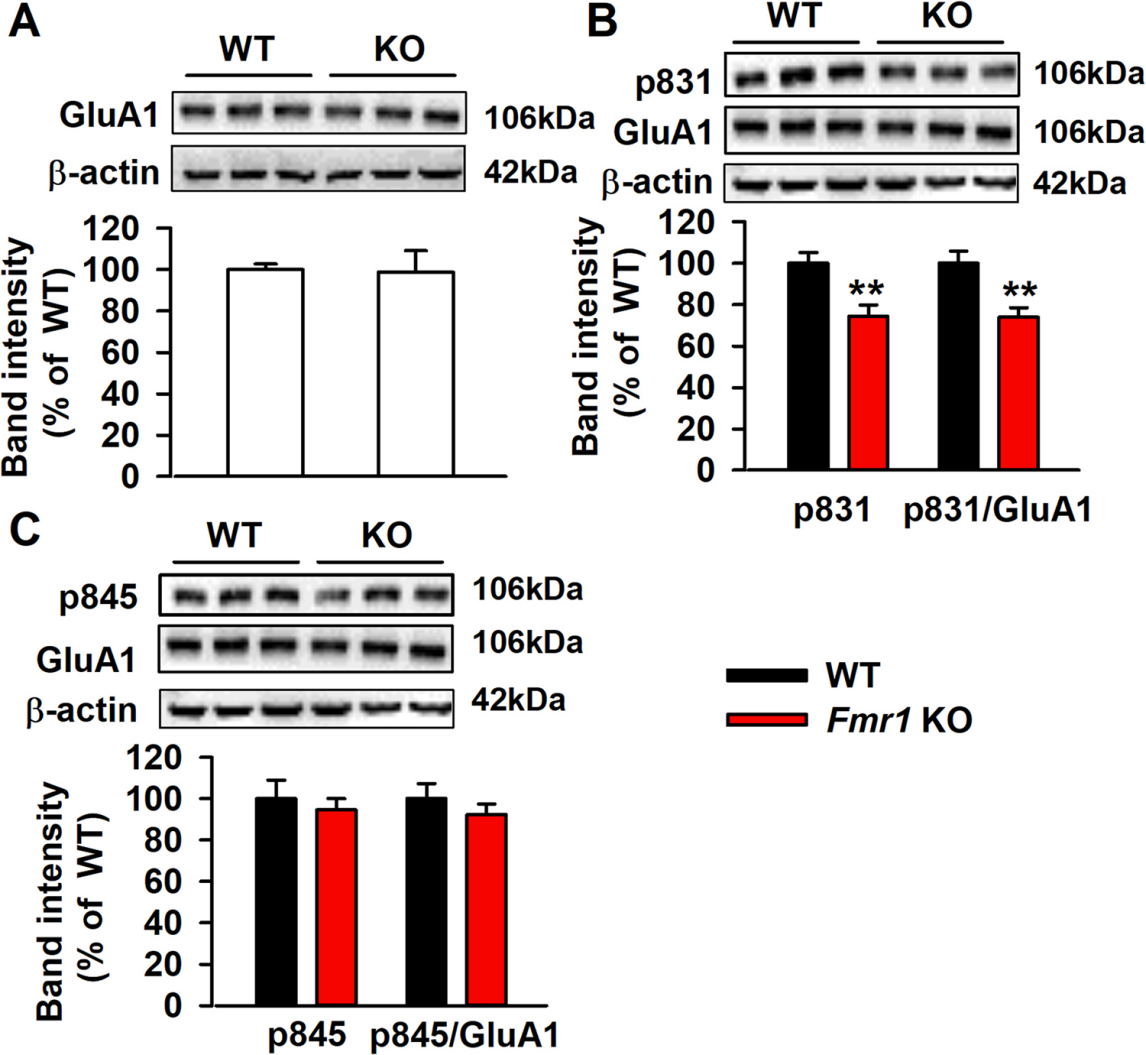
GluA1 subunit phosphorylation in mPFC. **A** No significant change of GluA1 in the mPFC between *Fmr1* WT and KO mice. **B** Significant decrease in phosphorylation of GluA1 at serine 831 (p831) in the mPFC of *Fmr1* KO mice as compared to WT mice. **C** No significant change of p845/GluA1 in the mPFC between *Fmr1* WT and KO mice. *n* = 6 in each group, **P < 0.01 vs WT control mice

Ca^2+^ entry via NMDARs leads to phosphorylation of serine831 (S831) and S845 of the GluR1 subunit of the AMPAR. Phosphorylation of serine residues is critical for synaptic insertion of the GluR1 receptor [25–27]. Induction of LTP is dependent on the NMDARs activation; however, expression of LTP is dependent on the AMPAR surface insertion and phosphorylation [28]. Levels of total GluA1 were similar between the WT and KO mice (Fig. 4A), whereas levels of phosphorylation of GluA1 at serine 831 (GluA1-p831) were lower in the *Fmr1* KO mice (Fig. 4B). Another phosphorylation site of GluA1 at serine 845 (GluA1-p845) had no difference between the WT and KO mice (Fig. 4C).

### Rescue of Impaired LTP by D-Asp in mPFC of Fmr1 KO Mice

**Fig. 5:**
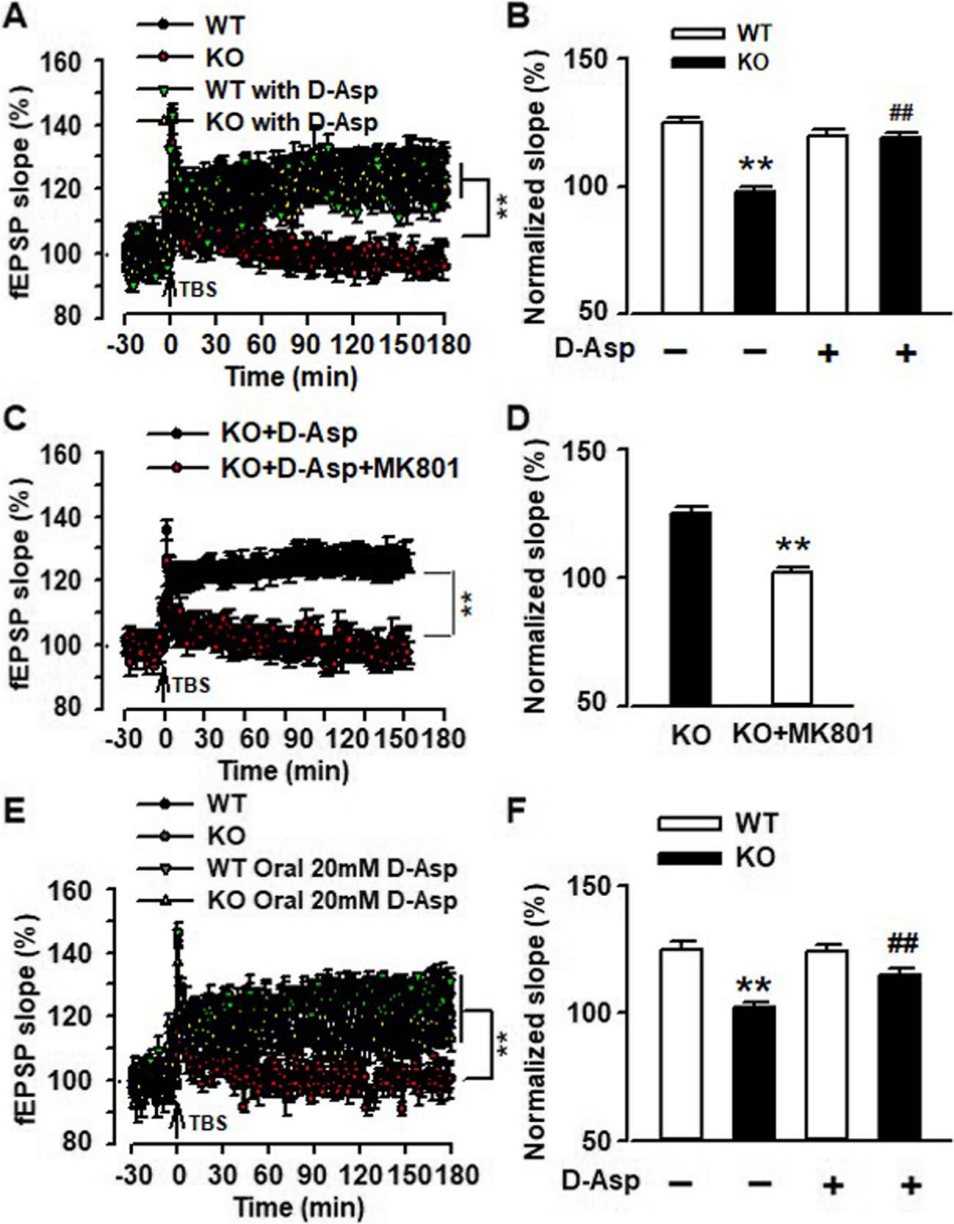
Rescue of LTP impairment by D-Asp perfusion in KO mice. fEPSPs were recorded under the MED64 recording system. **A**–**B** Perfusion of D-Asp (10 μM) did not alter LTP in WT slices (n = 8 slices/4 mice in each group); however, acute perfusion of D-Asp (10 μM) rescued the LTP induction in slices from KO mice (*n* = 10 slices/5 mice in each group). **C**–**D** LTP induction was abolished by MK801 (50 μM) in mPFC from *Fmr1* KO mice (*n* = 8 slices/3 mice in each group). **E**–**F** Male pups (8 weeks old) were used in the experiments. Chronic oral administration of D-Asp (20 mM) did not alter LTP in WT slices (WT: *n* = 6 slices/3 mice; WT treated with D-Asp: *n* = 8 slices/3 mice). It rescued the LTP induction in KO mice (*n* = 7 slices/3 mice in each group). **P <0.01 vs WT control mice; ^##^*P* < 0.01 vs KO control mice with two-way ANOVA-test

Impaired LTP was reported in the prefrontal cortex of *Fmr1* KO mice previously [19]. To investigate the effects of D-Asp on synaptic plasticity, we performed LTP induction in the mPFC slices from the mice without chronic drinking of D-Asp. Field excitatory postsynaptic currents (fEPSCs) were recorded in layers II/III of the mPFC with a MED64 system. Acute perfusion of D-Asp (10 μM, 10 min) did not alter LTP induction in WT slices (WT: 124.8 ± 2.3%, *n* = 8 slices/4 mice; WT perfused with D-Asp: 119.7 ± 3.2%, *n* = 8 slices/4 mice); however, it rescued the LTP induction in KO slices (KO: 97.9 ± 1.8%, *n* = 10 slices/5 mice; KO perfused with D-Asp: 119.2 ± 1.9%, *n* = 10 slices/5 mice; Fig. 5A and B). MK-801 (NMDAR antagonist, 50 μM) blocked the facilitation of LTP by D-Asp in *Fmr1* KO mice (MK801: 101.83 ± 3.87%, *n* = 7 slices/3 mice; D-Asp: 126.23 ± 3.16%, *n* = 6 slices/3 mice; Fig. 5C and D), indicating that LTP induction by D-Asp depends on NMDAR activation. Next, we determined whether chronic administration of D-Asp could restore LTP in KO mice. Pregnant female mice were orally administered D-Asp (20 mM) in drinking water, and their pups were fed with this modified drinking water as well. Male pups (8 weeks old) were used in the experiments. Oral administration of D-Asp restored LTP induction in the mPFC of *Fmr1* KO mice (KO: 97.86 ± 5.39%, *n* = 7 slices/3 mice; KO treated with D-Asp: 112.75 ± 4.29%, *n* = 7 slices/3 mice); however, additional D-Asp in the drinking water did not enhance LTP induction in WT mice (WT: 126.23 ± 3.16%, *n* = 6 slices/3 mice; WT treated with D-Asp: 123.74 ±5.43%, *n* = 8 slices/3 mice; Fig. 5E and F).

### Restored GluA1 Phosphorylation in mPFC of Fmr1 KO Mice

**Fig. 6:**
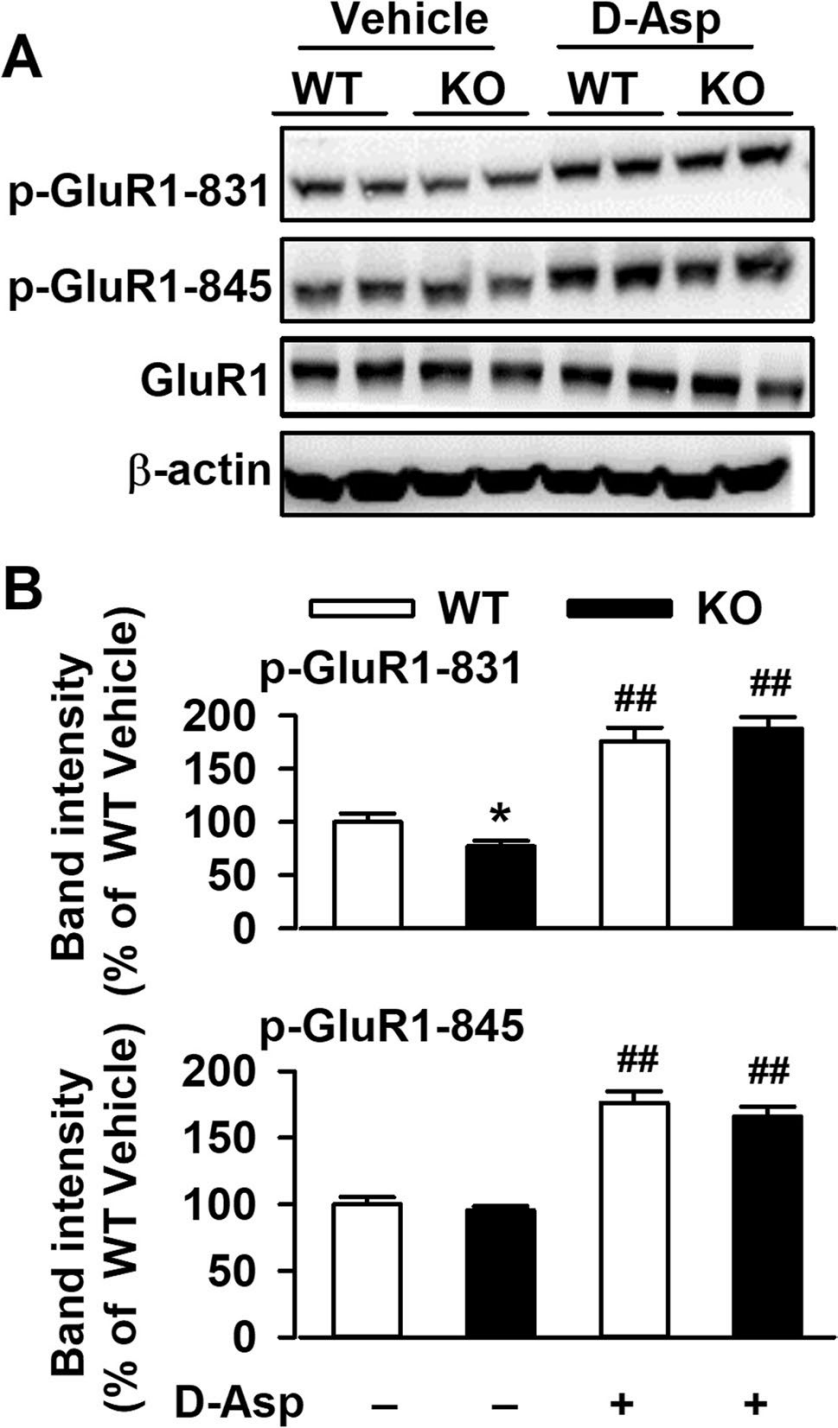
Effects of D-Asp oral administration on GluR1phosphorylation. **A** Representative Western blots for phosphorylation of GluR1 at S831, s845 and total GluR1 in mPFC from WT and Fmr1 KO mice. **B** Both of levels of phosphorylation of GluR1 at S831 and s845 were markedly increased in the mPFC of *Fmr1* KO mice with D-Asp oral administration. *n* = 6 in each group, **P <0.01 vs WT control mice; ^##^*P* < 0.01 vs KO control mice with two-way ANOVA-test

Oral D-Asp administration increased the levels of GluA1-p831 and GluA1-p845 in both of WT and KO mice (Fig. 6A). Even though levels of GluA1-p831 were lower in the KO mice, oral D-Asp administration restored it to the comparable levels of WT mice (Fig. 6B). The response of GluA1-p845 to oral D-Asp administration was similar between WT and KO mice.

### D-Asp Exposure Suppresses mGluR5 Signaling in Culture Cortical Neurons

**Fig. 7:**
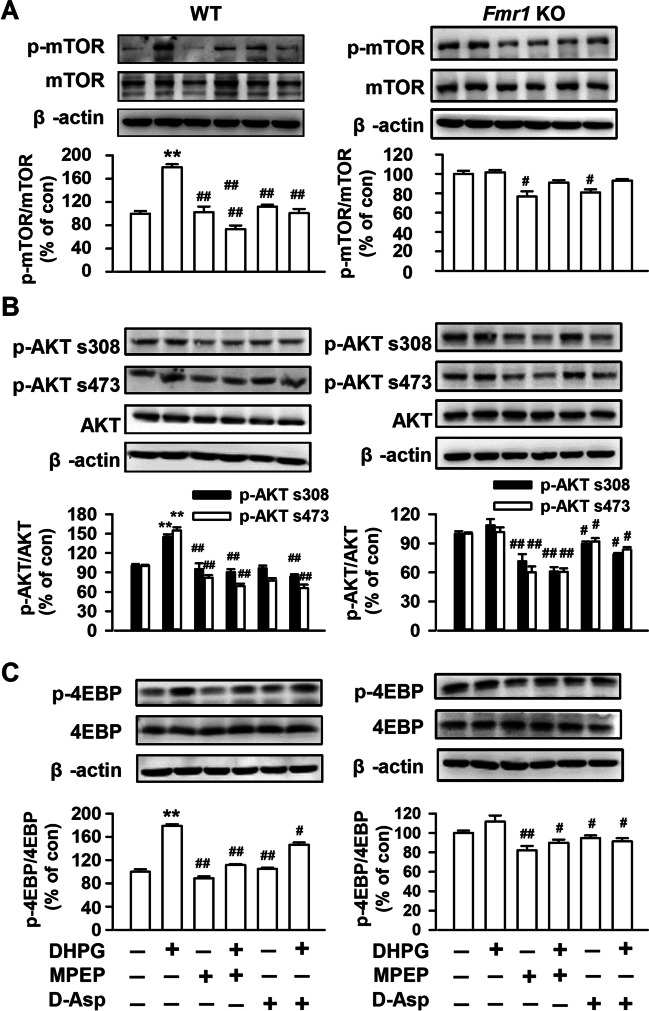
Effects of D-Asp on mTOR/4E-BP and AKT signaling in neural cultures. Primary cultures of cortical neurons of *Fmr1* WT and KO mice were pretreatment with saline, D-Asp (10 μM), or MPEP (50 μM) for 60 min, then cells treated with DHPG (100 μM for 5 min). **A** Representative Western blots for p-mTOR and mTOR proteins in cortical neuron cultures. Activation of mGluR5 with DHPG (100 μM) increased the levels of p-mTOR in WT cortical neurons, while no significant increase of p-mTOR expression after DHPG treatment in cortical neurons of *Fmr1* KO mice; pretreatment with MPEP (50 μM) or D-Asp (10 μM) abolished mTOR phosphorylation in WT. **B** Representative Western blots for total AKT, AKT phosphorylation at S308, and AKT phosphorylation at S473 in cortical neuron cultures. Activation of mGluR5 with DHPG (100 μM) increased the levels of phosphorylation of AKT at S308 and s473 in WT cultures but not in *Fmr1* KO cultures; however, pretreatment with MPEP (50 μM) or D-Asp (10 μM) abolished the increase of phosphorylation of AKT at S308 and s473 in both cultures of cortical neurons of WT and KO mice. **C** Representative Western blots for p-4E-BP and 4E-BP proteins in cortical neuron cultures. Activation of mGluR5 with DHPG (100 μM) increased levels of p-4E-BP significantly in WT cultures but did not induce prominent change in *Fmr1* KO mice; however, pretreatment with MPEP or D-Asp abolished p-4E-BP phosphorylation of both cortical neurons of *Fmr1* WT and KO mice. *n* = 5 dishes in each group, **P < 0.01 vs saline control; ^#^*P* < 0.05, ^##^*P* < 0.01 vs DHPG-treated group with one-way ANOVA-test

An exaggerated consequence of mGlu5-mediated signaling in the absence of FMRP has been proposed to trigger of many behavioral phenotypes of FXS [29]. Genetic or pharmacologic suppression of mGlu5 is sufficient to ameliorate a broad range of phenotypes in the KO mouse [7, 30–32]. Thus, we hypothesize that D-Asp rescues LTP due to regulating mGluR5 signaling. Activation of mGluR5 with DHPG (an mGluR5 selective agonist, 100 μM) for 5 min significantly induced phosphorylation of mTOR (p-mTOR, 180.0 ± 5.0% of control; Fig. 7A), AKT (p-AKT s308, 144.9 ± 4.3% of control; p-AKT s473, 155.2 ± 4.7% of control; Fig. 7B), and 4E-BP (p-4E-BP 178.9 ± 2.7% of control; Fig. 7C) in cultured WT neurons. However, pretreatment with D-Asp (10 μM) for 60 min abolished mTOR phosphorylation (101.1 ± 7.0% of control; Fig. 7A), AKT (p-AKT s308, 83.9 ± 3.4% of control; p-AKT s473, 65.5 ± 5.4% of control; Fig. 7B), and 4E-BP (146.4 ± 3.8% of control; Fig. 7C) induced by DHPG in WT neurons. Pretreatment with MPEP (50 μM), an mGluR5 selective antagonist, induced similar effects on the levels of p-mTOR, p-AKT, and p-4E-BP as D-Asp did. Activation of mGluR5 with DHPG for 30 min did not induced additional phosphorylation of mTOR (p-mTOR, 101.6 ± 2.4% of control; Fig. 7A), AKT (p-AKT s308, 108.7 ± 6.3% of control; p-AKT s473, 101.9 ± 4.7% of control; Fig. 7B), and 4E-BP (p-4E-BP 111.6 ± 6.3% of control; Fig. 7C) in cultured KO neurons. However, pretreatment of D-Asp (10 μM) alone for 60 min decreased the phosphorylation of mTOR (p-mTOR, 80.9 ± 3.2% of control; Fig. 7A), AKT (p-AKT s308, 89.4 ± 2.5% of control; p-AKT s473, 92.0 ± 3.4% of control; Fig. 7B), and 4E-BP (p-4E-BP 94.9 ± 2.5% of control; Fig. 7C) in cultured KO neurons. Furthermore, pretreatment with D-Asp (10 μM) for 60 min decreased the phosphorylation of AKT (p-AKT s308, 78.7 ± 1.5% of control; p-AKT s473, 83.7 ± 2.5% of control; Fig. 7B) and 4E-BP (p-4E-BP 91.2 ± 3.3% of control; Fig. 7C) in cultured KO neurons followed by DHPG treatment.

### Exogenous D-Asp Ameliorates Behavioral Deficits of Fmr1 KO Mice

**Fig. 8:**
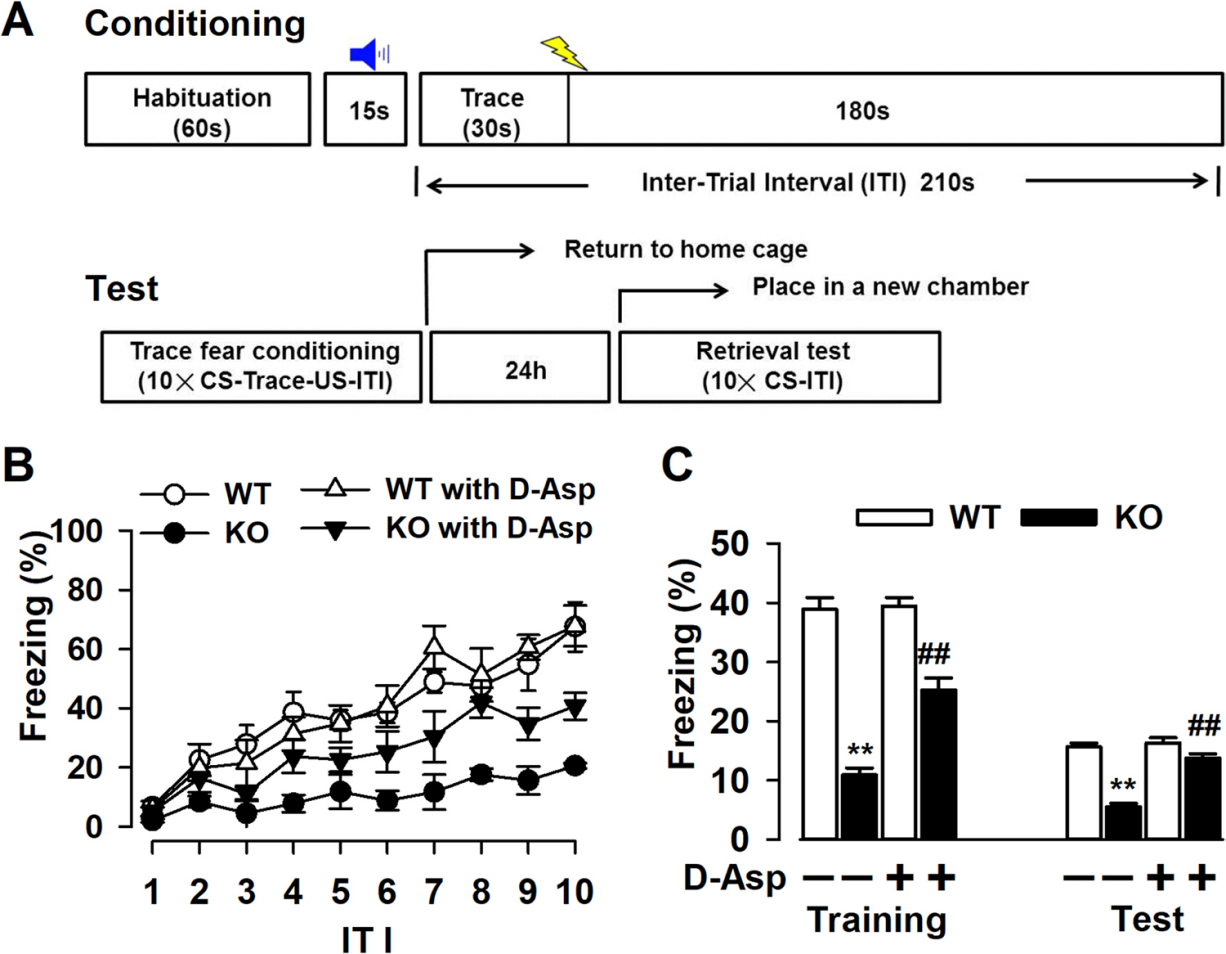
Chronic treatment of D-Asp improves the learning and memory of *Fmr1* KO mice. **A** The diagrammatic time course for trace fear memory training and testing. **B** *Fmr1* KO mice showed significantly reduced freezing during ITI-4 to ITI-10 as compared with *Fmr1* WT mice during the training phase. Supplement of D-Asp increased freezing time of KO mice during ITI4 and ITI-8 to ITI-10. **C** *Fmr1* KO mice exhibited less freezing time in the testing phase. Oral administration of D-Asp reversed this reduction in *Fmr1* KO mice. *n* = 6 in each group, **P < 0.01 vs WT control mice; ^##^*P* < 0.01 vs KO control mice with two-way ANOVA-test

Cognitive impairment is a core symptom of FXS. Cognitive defects of *Fmr1* KO mice were related to defects in attention, reflected by deficits in trace fear memory [22]. In the trace fear memory test, CS, an 80 dB white noise delivered for 15 s, was applied 30 s before (trace) the US, a 0.7 mA scrambled footshock. Mice were presented with 10 CS–trace–US trials with an ITI of 210 s. One day after training, mice received 10 CS–ITI trials in a novel chamber to test trace fear memory (Fig. 8A). Animal freezing times during these two sessions were recorded and analyzed by a computer. *Fmr1* WT mice successfully learned trace fear conditioning after 10 CS-ITI trails and had increased freezing throughout the training session (6.7 ± 2.1% freezing during ITI-1 vs 67.5 ± 8.3% freezing during ITI-10; Fig. 8B). Freezing time was also increased in *Fmr1* KO mice after 10 CS-US pairings (2.3 ± 0.5% freezing during ITI-1 vs 20.6 ± 1.1% freezing during ITI-10; Fig. 8B). However, *Fmr1* KO mice had significantly less freezing time compared with WT mice in the training course. Furthermore, supplementation of D-Asp increased freezing time in *Fmr1* KO mice during the test (KO control: 17.6 ± 2.0% freezing during ITI-8; KO with D-Asp supplement: 41.9 ± 5.1% freezing during ITI-8; Fig. 8C). Additional D-Asp did not affect trace fear memory in WT mice. Thus, *Fmr1* KO mice are deficient in acquiring trace fear memory during training and in expressing trace fear memory during testing on the following day. Chronic treatment with D-Asp partially rescued learning and memory deficits in *Fmr1* KO mice.

### Exogenous D-Asp Improves the Locomotor Activity of Fmr1 KO Mice

**Fig. 9:**
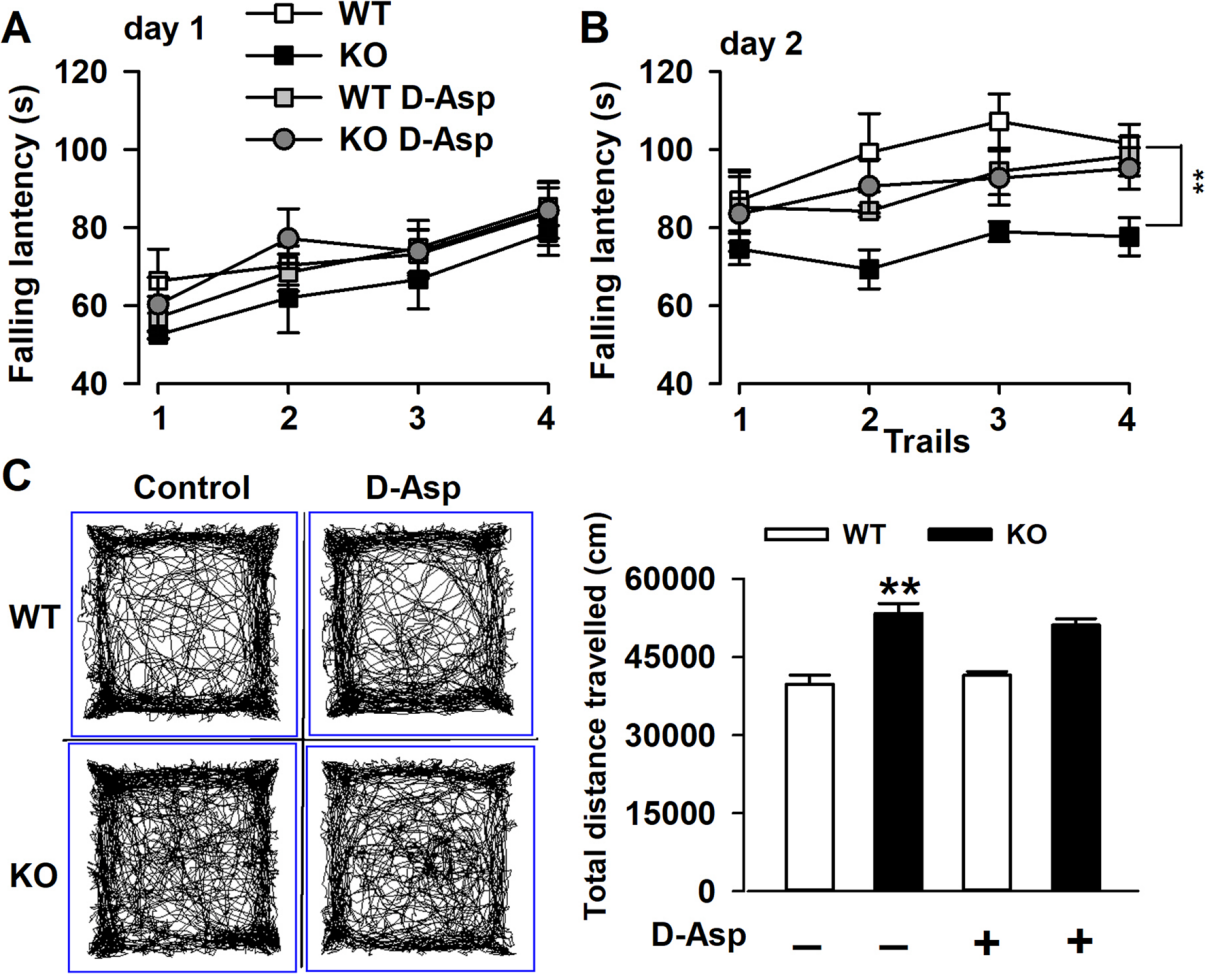
Chronic treatment of D-Asp improves motor coordination ability of *Fmr1* KO mice. **A-B** Motor coordination was tested by the rotarod test. Oral supplementation of D-Asp improved motor coordination of KO mice as compared to their control littermates. **B-C** Left: sample traces of locomotor activity in the open-field test. Right: *Fmr1* KO mice exhibit hyperactivity, but oral administration of D-Asp did not alter the locomotor activity. *n* = 6 in each group, **P < 0.01 vs WT control mice; #*P* < 0.05, ^##^*P* < 0.01 vs KO control mice with two-way ANOVA-test

*Fmr1* KO mouse is significantly impaired in motor coordination and shows hyperactivity [33].To examine motor coordination and skill acquisition, performance on the rotarod was tested. Oral supplementation of D-Asp significantly improved motor coordination of KO mice across eight trials over 2 days in rotarod test as compared to their control littermates (Fig. 9A and B), but additional D-Asp failed to alter KO mice hyperactivity (Fig. 9C and D).

## Discussion

Here, we report that a lack of FMRP increases the expression of DDO, a specific enzyme for D-Asp metabolism in a FXS mouse model. High DDO reduced D-Asp in the mPFC area. Moreover, chronic oral administration of D-Asp rescued LTP impairment and abnormal behavioral phenotypes in the FXS model mouse offspring.

NMDARs are diverse in their subunit composition, subcellular localization, and biophysical and pharmacological properties; however, their activation requires the binding of a co-agonist that has long been thought to be glycine. Recent research indicates that D-serine is the preferential co-agonist for a subset of synaptic NMDARs in many areas of the adult brain [34]. Glial derived D-serine has been reported to control NMDAR activity and synaptic memory [35]. Recent work suggests that treatment with NMDAR co-agonists (glycine or D-serine) independently rescue impairments in NMDAR-dependent synaptic plasticity in the dentate gyrus of *Fmr1* KO mice [13]. However, much less is known about the role of D-Asp in mammals. L- and D-Asp are putative NMDAR agonists, mimicking most actions of glutamate on NMDARs by sharing the same binding site [36]. Increase of brain D-Asp facilitates the maintenance of hippocampal LTP and prevents synaptic depotentiation [15, 37]. In specific regions of DDO^-/-^ mice, such as the hippocampus, striatum, cortex, cerebellum, and olfactory bulbs, D-Asp is ~10- to 20-fold greater than that in corresponding wild-type brain areas [38]. Morris water maze performance and contextual fear conditioning in DDO^-/-^ mice are improved compared to wild-type animals. Here, we report that DDO protein in the mPFC area of *Fmr1* KO mice increased by 1.5-fold compared to WT mice. FMRP is an mRNA-binding protein that controls translation of targeting proteins [2, 3]. Loss of FMRP in *Fmr1* KO mice may thus cause over-expression of DDO, which increases the metabolism of D-Asp in the brain. This finding is consistent with previous reports that oral administration of D-Asp increases endogenous D-Asp in the hippocampus by 2- to 5-fold compared to the same brain areas of untreated mice [17].

GluA1 phosphorylation at Ser831 leads to an increase in the conductance of AMPARs during the LTP induction [39]. Although phosphorylation at the Ser845 site is more critical for LTP expression, considering that LTP is reduced when lacking both phosphorylation sites, but is normal when only lacking one, either Ser831 or Ser845 alone can support LTP. Thus, lacking both sites compromises LTP stability. In cultured neurons, basal GluR1 and phosphorylation at Ser845 site were similar between *Fmr1* KO and WT mice. However, GluA1 phosphorylation at Ser831was decreased in KO mice. Treatment with D-Asp increased the phosphorylation of GluA1 at Ser 831 and Ser845 site in *Fmr1* KO neurons. It suggests an impairment of D-Asp activating signaling pathways in the basal conditions of KO neurons. Consistent with GluR1 phosphorylation, acute perfusion of D-Asp in brain slices or chronic oral administration of D-Asp could facilitate LTP induction in mPFC of *Fmr1* KO mice.

Recently, the mGluR theory of FXS has gained significant support [29]. This theory posits that dysregulated mGluR1/5-dependent synaptic plasticity contributes to FXS pathology, with significant enhancement of mGluR-dependent LTD in the hippocampus [40] and decreased cortical LTP [41]. In contrast to exaggerated mGluR5, the NMDA receptor is reported to be hypofunctional in the dentate gyrus and causes impaired context discrimination in adult *Fmr1* KO mice [42, 13]. Thus, the imbalance between low NMDAR function and enhanced mGluR5 function may be critical to the pathophysiology of FXS. As a specific agonist for regulation of NMDARs, increased D-Asp in the brain rescues hippocampal age-related synaptic plasticity deterioration in mice [17]. Therefore, elevation of brain D-Asp could reverse the imbalance between NMDARs and mGluR5, perhaps serving as a therapeutic target for FXS.

Activation of mGlu5 leads to activation of Ga_o/q_, intracellular Ca^2+^ mobilization, and stimulation of PLC and PI3K [4]. Down-stream signaling events including activation of mTOR and GSK3β modulate synaptic protein translation and transcription [43]. Chronic oral administration of D-Asp decreased the upregulation of mTOR, 4E-BP, and AKT signaling in FXS mice. Facilitation of LTP induction by exogenous D-Asp suggests that insufficient D-Asp contributes to learning and memory deficit of *Fmr1* KO mice.

## Conclusion

In this study, we found chronic oral administration of D-Asp reversed behavioral deficits of cognition and locomotor coordination in *Fmr1* KO mice. The therapeutic action of D-Asp was partially through regulating functions of NMDARs. Thus, supplement of D-Asp may benefit for synaptic plasticity and behaviors in *Fmr1* KO mice and offer a potential therapeutic strategy for FXS.

## Data Availability

All data generated or analyzed during this study are included in this published article.

## Abbreviations

ACC: Anterior cingulate cortex
D-Asp: D-Aspartate
DDO: D-Aspartate oxidase
FXS: Fragile X syndrome
FMRP: Fragile X mental retardation protein
*Fmr1*: Fragile X mental retardation 1 gene
HPLC: High-performance liquid chromatography
LTP: Long-term potentiation
NMDAR: N-Methyl-D-aspartate receptor
mPFC: Medial prefrontal cortex
